# Giving away used injection equipment: missed prevention message?

**DOI:** 10.1186/1477-7517-7-2

**Published:** 2010-02-09

**Authors:** Carol Strike, Daniel Z Buchman, Russell C Callaghan, Cass Wender, Susan Anstice, Brian Lester, Nick Scrivo, Janine Luce, Margaret Millson

**Affiliations:** 1 Centre for Addiction and Mental Health, 33 Russell Street, Toronto, Ontario Canada; 2 Dalla Lana School of Public Health, University of Toronto, Toronto, Ontario Canada; 3 My Sisters' Place - Transitional Support Centre for Women who are Homeless or at Risk of Homelessness, London, ON, Canada; 4 Factor-Inwentash Faculty of Social Work, University of Toronto, Toronto, Ontario Canada; 5 Counterpoint Needle Exchange, AIDS Committee of London, London, Ontario, Canada

## Abstract

**Background:**

Our objective was to examine factors associated with distributive injection equipment sharing and how needle exchange programs (NEPs) can help reduce distributive sharing among injection drug users (IDUs).

**Methods:**

145 English speaking Canadian IDUs ages 16 years and over who had injected in the past 30 days were recruited for a cross-sectional survey. Participants were asked about their socio-demographic characteristics, HIV risk behaviours, social support, drug treatment readiness, program satisfaction, health and social service use and NEP drug use. Bivariate statistics and logistic regression were used to characterize the population and examine correlates of sharing behaviour.

**Results:**

More IDUs reported distributive sharing of cookers (45%) than needles (36%) or other types of equipment (water 36%; filters 29%; swabs 8%). Regression analyses revealed the following factors associated with distributing used cookers: a history of cocaine/crack injection, an Addiction Severity Index (ASI) score indicative of a mental health problem, and older than 30 years of age. Factors associated with giving away used water included: male, injected methadone, injected other stimulants and moved 3+ times in the past 6 months. Factors associated with giving away used filters included: injected cocaine/crack or stayed overnight on the street or other public place. Factors associated with giving away swabs included: an ASI mental health score indicative of a mental health problem, and HCV negative status.

**Conclusions:**

Our findings show that more IDUs give away cookers than needles or other injection equipment. While the results showed that correlates of sharing differed by piece of equipment, each point to distributive sharing by the most marginalized IDUs. Targeting prevention efforts to reduce equipment sharing in general, and cookers in particular is warranted to reduce use of contaminated equipment and viral transmission.

## Background

In Canada, the prevalence of HIV and Hepatitis C among injection drug users remain public health concerns [1,2]. Recent evidence shows that receptive (taking used needles from someone else) and distributive (giving used needles to someone else) sharing among IDUs has declined [3-7]. However, there are also risks associated with re-using drug injection equipment such as cookers, water, filters and alcohol swabs [8-13]. Many studies do not inquire about each piece of equipment independently and/or distinguish between receptive, distributive or communal equipment sharing (i.e., using a communal set of equipment to mix and apportion a drug solution to more than one person) [12]. This is important because evidence suggests that IDUs are more likely to report equipment sharing than needle sharing [3,7,14]. As well, some studies report that IDUs are more likely to distribute than receive used injection equipment [6,7].

The potential for viral transmission can occur at various stages of drug preparation and involve multiple pieces of equipment. Drug preparation processes vary by type of drug and location. In North America to prepare a drug for injection, it must be dissolved with water in a cooker or spoon. To dissolve some drugs, the water needs to be heated. Once the drug is dissolved, the solution is drawn through a filter, up into a needle, and into a syringe. Then, the solution is injected back through the needle through skin that may or may not have been cleaned with an alcohol swab, and into a vein [12,15]. If any of these pieces of equipment used to prepare the drugs, not just the needle and syringe, are contaminated, there is a potential for viral transmission.

Another important observation from existing research is that the frequency with which IDUs report sharing varies by piece of equipment. Of all types, cookers are the most commonly shared piece of equipment [8,11,14-17]. Studies show varied rates of sharing cookers ranging from 65% to 84% [6,10]. As well, Hunter and colleagues reported cooker sharing was common among IDUs who did not share needles. Sharing of cookers has been linked with high perceived risk or the inevitability of acquiring an HIV infection [18]. The filters used to remove debris from drug solutions are also shared by IDUs. The frequency with which this has been reported varies from 50% to 77% [10,19,20]. Reports of sharing the water used to mix drug solutions and rinse equipment vary from a low of 15% to over 83% [6,10,11,7,19,20]. In comparison with other types of equipment, fewer IDUs report sharing alcohol swabs [15]. While research shows sharing varies by pieces of equipment, studies do not distinguish between receptive and distributive practices. Thus, equipment sharing, particularly distributive sharing, may not have received as much attention in public health research and programming as necessary to remove contaminated equipment from circulation and reduce the potential for viral transmission.

Given the limited examination of distributive sharing practices by each piece of equipment, this manuscript focuses on answering two questions: what factors are associated with distributing specific pieces of injection equipment, and how can needle exchange programs (NEPs) help reduce distributive sharing among IDUs? Giving another person previously used injecting equipment is labeled in the literature as 'donating' or 'donor sharing' [17], 'lending' [21,22], 'distributive sharing' [23,24], and 'passed on' [25,26]. We use the term 'distributive sharing' because 'sharing' and 'lending' suggest that the equipment is returned and this may not always reflect what transpires.

## Methods

From September 2006 to January 2007, IDUs in London, Ontario were invited to participate in a cross-sectional survey regarding their drug use. The IDU Outreach Coordinator and Community Co-Investigators based at the Counterpoint Needle Exchange Program in London, Ontario advertised the study by word of mouth and printed flyers. The inclusion criteria were being 16 years of age or older, English speaking, and having injected in the past 30 days. IDUs expressing an interest in the study were introduced to the research staff who explained the study's purpose. Potential participants who were introduced to the research staff were invited to an interview room where the study was explained in more detail. All participants who were introduced to the staff provided informed consent. Clients who were too intoxicated to give informed consent were asked to return on another day. Participants were compensated $20. The Research Ethics Board at the Centre for Addiction and Mental Health and the Board of Directors at the AIDS Committee of London which administers the NEP approved the study protocol. This study employed a community based research design wherein academic researchers, NEP service providers and client representatives shared responsibility for the conduct of the study, analyses and interpretation of results.

We used a stratified, quota sampling technique to maximize the representativeness of the sample in terms of gender in relation to the local IDU population (i.e., 70% male and 30% female) because there is no sampling frame for local IDUs. Participants were recruited until the quota in each stratum was reached. Using a structured questionnaire, participants were asked questions about their socio-demographic characteristics, injection and sexual risk behaviours, perceived social support, drug treatment readiness, program satisfaction, housing status, income and employment, and health and social service use, and completed and the self-report version of the Addiction Severity Index [27]. To measure distributive sharing, we asked the following question for each piece of equipment: in the past 6 months, did anyone else use the {*insert piece of equipment*} that you had already used? This includes your sex partner(s).

To characterize the population and examine correlates of behaviour, we used univariate and bivariate statistical tests and logistic regression. The analyses were performed using SPSS 14.0 and conducted in two steps. First, chi-squared tests were used to examine the strength of the associations between distributive sharing behaviours and independent variables that have been identified in the literature: age under 30 versus 30 years or more, gender, number of housing moves in the past 6 months (0-2 versus 3 or more); stayed outside at least one night in the past 6 months, types of drugs injected (i.e., prescription opiates/heroin; speedballs, methadone, crack or powder cocaine; crystal methamphetamine; other stimulants), injecting outdoors in the past 6 months, injecting at a shooting gallery or dealer's place in the past 6 months, ASI psychiatric composite score ≥ 0.4, perceived risk of HIV infection and HCV infection status. In response to evidence of variation in sharing by piece of equipment, we performed the analyses separately for each piece of equipment. To build the model, we used standard criteria where independent variables in the bivariate analyses reaching significance of ≤ 0.250 were entered into the logistic regression model [28]. We used forward stepwise logistic regression to test associations between distributive sharing and independent variables.

## Results

We interviewed 145 current IDUs. Of the participants, 72.4% were male which reflects the known gender distribution of clients accessing Counterpoint, the local NEP (Table 1). Most participants were over age 30 (79.2%) and the majority had never been married (54.5%). Many had not completed high school (52.8%) and just over half received social assistance income (52.1%). Many participants moved 3 or more times in the past 6 months.

**Table 1:** Socio-demographic characteristics

		n	%
Gender	Male	105	72.4
	
	Female	40	27.6

Age	≤ 29	30	20.8
	
	> 30	114	79.2

Marital status	Never married	79	54.5
	
	Separated or divorced	43	29.7
	
	Married	17	11.7
	
	Widowed	6	4.1

Completed education	Less than high school	76	52.8
	
	High school or more	68	47.2

Receiving social welfare	No	69	47.9
	
	Yes	75	52.1

Number of housing moves in last 6 months	0-2	71	54.2
	
	3+	60	45.8
	
	Mean	131	6.24

More participants distributed cookers (45%) than used needles (36%). Distribution of other pieces of equipment was also reported: 36% water, 29% filters, 8% swabs. Re-use of other equipment was also reported: 19% water, 18% filters, 6% swabs. Many also reported re-using someone's cooker (37%). When asked, 21% reported using a needle that had been used by someone else.

Table 2 presents a summary of the variables associated with distribution of at least one type of equipment and with a *p value *of ≤ 0.250. None of the variables we tested reached this level of significance for all types of equipment but many were associated with at least two distribution variables. Nevertheless, any variable associated with at least two distribution variables was included in all logistic regression analyses.

**Table 2:** Distributive sharing of equipment by selected demographic, health and past 6 month drug use behaviour variables

Variables		% of total sample	% (n) reported distributing to someone else			
			
			cooker/spoon	water	filter	swabs
Age	≤ 29	20.8	62.1 (18)*	48.3 (14) *	36.7 (11)	0.0 (0)
	
	≥ 30	79.2	41.7 (43)*	33.0 (34)*	27.2 (28)	10.6 (11)*

Sex	Male	72.4	45.3 (43)	33.3 (32) *	26.0 (25)*	8.3 (8)
	
	Female	27.6	50.0 (19)	45.9 (17) *	36.8 (14)*	7.9 (3)

Self-reported Hepatitis C viral status	Positive	53.1	56.1 (32)	54.3 (25)	59.5 (22)	3.0 (2)*
	
	Negative	46.9	43.9 (25)	45.7 (21)	40.5 (15)	14.3 (8)*

ASI Psychiatric Composite Score	< 0.4	68.3	41.3 (38)*	32.6 (30)*	25.8 (24)*	5.4 (5)*
	
	≥ 0.4	31.7	24 (60.0)*	47.5 (19)*	37.5 (15)*	14.6 (6)*

Number of housing moves in past 6 months	0-2	54.2	43.3 (29)	29.9 (20)*	26.9 (18)	10.1 (7)
	
	3+	45.8	51.8 (29)	44.6 (25)*	32.1 (18)	7.3 (4)

Stayed outside (e.g., street, park, stairwell, vehicle etc.) for at least one night	Yes	51.0	61.3 (38)*	61.2 (30)*	66.7 (26)*	54.5 (6)
	
	No	49.0	41.4 (29)*	45.8 (38)*	44.7 (42)*	45.5 (5)

Used speedballs - heroin and cocaine and/or Ritalin and methadone	Yes	28.5	51.4 (19)	48.6 (18)*	40.5 (15)*	13.5 (5)*
	
	No	71.5	44.8 (43)	32.3 (31)*	24.7 (24)*	6.2 (6)*

Injected Methadone - prescribed and non-prescribed	Yes	27.8	61.1(22)*	52.8 (19)*	38.9 (14)*	13.5 (5)*
	
	No	72.2	41.2 (40)*	30.9 (30)*	25.5 (25)*	6.2 (6)*

Injected cocaine and/or crack	Yes	68.1	53.3 (48)*	41.1 (37)*	34.1 (31)*	9.9 (9)
	
	No	31.9	32.6 (14)*	27.9 (12)*	18.6 (8)*	4.7 (2)

Injected other stimulants and amphetamines (Ritalin, monster, crank, Benzedrine, Dexedrine, Preludin, Methamphetamine, Speed, Ice, Crystal)	Yes	53.1	51.4 (37)*	47.2 (34)*	36.1 (26)*	8.3 (6)
	
	No	46.9*	40.0 (24)*	25.0 (15)*	19.7 (12)*	8.2 (5)

Used (non-inject) other sedatives, hypnotics, and tranquilizers	Have Used	52.8*	59.7 (37)	57.1 (28)	56.4 (22)	63.6 (7)
	
	Never Used	47.2*	40.3 (25) *	42,9 (21)	43.6 (17)	36.4 (4)

Injected outdoors	Yes	78.2*	83.9 (52)	39.6 (42) *	32.7 (35) *	81.8 (9)
	
	No	21.8*	16.1 (10)	25.9 (7) *	14.8 (4) *	18.2 (2)

* Indicative of a statistical difference of ≤ 0.250

Results from logistic regression analyses revealed that variables independently associated with distributive sharing varied by equipment type (Table 3). IDUs with a history of cocaine/crack injection had the highest odds of distributing cookers (AOR = 5.67), followed by an ASI composite score ≥ 0.4 (AOR = 3.257). IDUs aged 30 and over (AOR = 0.344) were considerably less likely than younger IDUs to distribute cookers.

**Table 3:** Variables independently associated with distributive sharing by type of equipment

Cooker/Spoon	Adjusted ORs	95% C.I	p
Injected cocaine and/or crack in the last 6 months	5.670	1.317 - 7.775	.001

ASI Psychiatric Composite Score (≥ 0.4)	3.257	1.29 - 8.220	.012

Age (> 30)	.344	0.122 - 0.971	.044

**Water**			

Injected methadone (prescribed/non-prescribed) in the past 6 months	3.116	1.199 - 8.093	.020

Injected other stimulants and amphetamines in the last 6 months	2.555	1.051 - 6.209	.038

Male	2.835	1.092 - 7.362	.032

Moved 3 or more times in the past 6 months	2.693	1.109 - 6.542	.029

**Filter**			

Injected cocaine and/or crack in the last 6 months	3.224	1.100 - 9.456	.031

Stayed outdoors/public place overnight in the past 6 months	2.479	1.048 - 5.866	.039

***Swabs***			

Hepatitis C Status (positive)	0.086	0.015 - 0.490	.006

ASI Psychiatric Composite Score (≥ 0.4)	5.648	1.217 - 26.221	.027

The findings were somewhat different for distributing injection water. The odds were higher for IDUs who had a history of injecting methadone (AOR = 3.116) and other stimulants (AOR = 2.555). Men (AOR = 2.835) were more likely than women to distribute water as were IDUs who moved 3 or more times (AOR = 2.693).

Injection of cocaine/crack (AOR = 3.224) and having spent at least one night on the street or other public place (AOR = 2.479) were independently associated with distributing filters. The odds of distributing alcohol swabs were lower for IDUs who self-reported being HCV positive (AOR = 0.086), but higher for those with an ASI composite score ≥ 0.4 (AOR = 5.648).

## Discussion

Our data demonstrate that many IDUs distributed their used injection equipment, and used cookers in particular. Other Canadian data indicates that approximately one-third of IDUs loaned their equipment to someone else [29]. However, this proportion varies across Canadian communities: from 23.4% in Quebec City to 46.8% in Regina [29]. Most IDUs (83.8%) who loan equipment to someone else do not do so every time (range 65.0% in Regina to 96.8% in Victoria) [28]. However, 14.7% of IDUs who loaned equipment said that they always did so (range: 3.2% in Victoria to 33.3% in Regina) [29].

Our findings and the accumulation of evidence from many jurisdictions demonstrates a differential habit of distributing versus receiving used injection equipment [3,6,7,30]. These findings lead one to question why do more IDUs report distributive versus receptive sharing of used injection equipment? This finding could be a realistic portrayal of behaviour or reflect the reluctance of IDUs to admit behaviour that conflicts with the prevention messages they receive from workers - 'don't prepare or inject drugs with previously used equipment'. However, Gossop suggests that 'the sharing of spoons and water does not have such an intimate or intrusive quality and the risks associated with sharing such types of injecting paraphernalia have been largely omitted from prevention messages" (p. 656) [14]. Consequently, some IDUs may perceive messages about sharing to be limited to needles and syringes and not other injecting equipment [8,31,32]. A study of heroin injecting networks provides some support for this hypothesis insofar as it was found that 67% of study participants reported mixing their drug solution with water that had been previously used to rinse used syringes [32]. As well, 86% reported using a shared cooker but only 22% reported sharing syringes [32]. Distributing used equipment may also reflect a tangible and intangible exchange relationship. IDUs may get something in return for giving away used equipment, such as drugs, trust, companionship, sex, friendship, or expectation of future reciprocity.

Our analyses showed that male IDUs who are older than 30 years, use stimulants and/or amphetamines, have concurrent mental health problems and whose housing is unstable are more likely than other IDUs to distribute equipment. These findings are consistent with those from other jurisdictions [33-35]. Our findings concerning the association between the distribution of cookers and swabs and mental health problems complement a recent meta analysis which found an association between depression and needle sharing [36]. We also found that associations differ by type of equipment. The reason for these differences is unclear. Examination of the bivariate associations shows some consistency (e.g., ASI score, staying outside overnight, injecting stimulants) but also some variation across the pieces of equipment and the characteristics of the distributers. Differences in the proportions of IDUs reporting distributive or receptive sharing by equipment and characteristics have been reported by others. These findings suggest the possibility of different drivers of distributive sharing and/or the influence of a small sample size on the logistic regressions. In the future, it will be important to determine if there are different drivers of distributive sharing and appropriate responses. Nevertheless, the findings point towards the need to ensure interventions reach those most likely to engage in distributive sharing, including older males and stimulant users. As well, our analyses suggest that IDUs who have severe addiction, who live with mental health problems and in very impoverished circumstances who may need extra supports to ensure they can benefit from HIV prevention programs. These findings also support a need to tie interventions aimed a reducing risk behaviours to intervention aimed towards improving mental health.

A cornerstone of NEPs involves routine collection and disposal of contaminated needles/syringes [15]. Our findings suggest the need for increased efforts to collect and dispose of injection equipment, particularly cookers. These efforts are warranted because studies suggest a narrow window of opportunity to prevent infections among injectors [9]. This window is particularly short for HCV infection and all efforts to lessen the re-use of equipment are necessary for prevention of viral transmission. Results from an evaluation of a peer-mentoring behavioural intervention suggest that transmission behaviours can be reduced [37]. These findings show a link between reduced risk behaviour and newly acquired knowledge of the risks of re-using other injection equipment and improved self-efficacy to avoid risk [37]. Reinforcing knowledge and skill development focused specifically on other injection equipment represents an intervention that can be further enhanced by NEP workers. As well, extending recommendations for safe disposal of needles to all injection equipment and improving awareness and convenience of safe disposal methods for equipment may improve disposal behaviours [15,38]. Targeting social networks may also be an important and effective means of changing equipment related behaviours [39].

Lack of a sampling frame for this population meant that we could not randomly sample. However, the data were collected using a stratified convenience sampling method to ensure that the sample reflected the gender division within this population. In terms of the age distribution of the population, anecdotal evidence from the NEP suggests that our sample reflects that of the local IDU population. Our findings were corroborated by the IDU co-investigators on our team who noted that the findings were consistent with their personal experiences and their knowledge of drug using behaviours in the local IDU community. IDU populations vary over time and place and the generalizeability of our findings and others face similar limitations.

## Conclusions

Recurrent findings that large proportions of IDUs distribute used equipment leads one to question whether prevention programs have focused so much on receptive sharing that they have inadvertently neglected to communicate the risks associated with distributive sharing. Our second question was, 'what can NEPs do to reduce distributive equipment sharing among IDUs?' Using our data, our multidisciplinary team including academic, service and drug use experts developed nine key messages for NEPs. To help reduce distribution of used injection equipment, NEPs can:

• Speak with clients about the risks of giving away equipment and the benefits of disposal

• Distribute educational materials about the risks of distributing other injection equipment

• Encourage clients to bring ALL used equipment to the NEP and any partner locations that will dispose of equipment

• Give clients extra sterile supplies to distribute to other IDUs

• Develop peer-based education and equipment distribution programs that target social networks

• Distribute biohazard waste containers

• Dispose of used equipment and biohazard containers

• Provide multiple and convenient locations for IDUs to dispose of used equipment

• Coordinate NEP education efforts and equipment distribution with agencies providing housing, mental health and other services to IDUs

## Competing interests

The authors declare that they have no competing interests.

## Authors' contributions

CS, SA, CW, BL, NS, JL, RS, PM and community co-investigators contributed to the design and coordination of the study. All authors helped to draft the manuscript. CS, DB and RC conducted the statistical analyses and completed the manuscript. All authors read and approved the final manuscript.
